# Clinical significance of redox effects of Kampo formulae, a traditional Japanese herbal medicine: comprehensive estimation of multiple antioxidative activities

**DOI:** 10.3164/jcbn.17-59

**Published:** 2017-12-12

**Authors:** Aki Hirayama, Shigeru Oowada, Hiromu Ito, Hirofumi Matsui, Atsushi Ueda, Kazumasa Aoyagi

**Affiliations:** 1 Center for Integrative Medicine, Tsukuba University of Technology, 4-12-7 Kasuga, Tsukuba, Ibaraki 305-8521, Japan; 2 Asao Clinic, 1-8-10 Manpukuji, Kawasaki, Kanagawa 215-0004, Japan; 3 Faculty of Medicine, University of Tsukuba, 1-1-1 Tennodai, Tsukuba, Ibaraki 305-8575, Japan; 4 Tsukuba University Hospital Hitachi Medical Education and Research Center, 2-1-1 Jyonancho, Hitachi, Ibaraki 317-0077, Japan

**Keywords:** Kampo, traditional Japanese herbal medicine, hydroxyl radical, multiple radical scavenging activity, *Bupleuri Radix*, *Rehmanniae Radix*

## Abstract

To clarify the clinical significance of the redox-controlling effects of Kampo, a traditional Japanese herbal medicine, we determined the scavenging activities of various reactive oxygen species in clinically used Kampo formulae using an electron spin resonance-based technique. Formulae containing *Rhei Rhizoma* (i.e., mashiningan and daiobotanpito) showed high scavenging activity against the alkoxyl radical, and crude extract quantity was significantly correlated with scavenging activity. Hydroxyl radical scavenging activity was positively correlated with the quantity of *Zingiberis Rhizoma*. Strong hydroxyl radical scavenging activity was also found in formulae containing both *Bupleuri Radix* and *Scutellariae Radix*, a widely used anti-inflammatory combination. Formulae containing a clinically common combination of *Scutellariae Radix*, *Coptidis Rhizoma*, and *Phellodendri Cortex* induced high superoxide scavenging activity. Singlet oxygen scavenging activity was high in formulae containing *Bupleuri Radix* and *Glycyrrhizae Radix*. In contrast, formulae containing *Rehmanniae Radix* showed generally low reactive oxygen species scavenging activities, and the quantity of *Rehmanniae Radix* was negatively correlated with hydroxyl radical and singlet oxygen scavenging activities. These results indicate that the antioxidative effects of Kampo formulae are not uniform but complexly varied against multiple reactive oxygen species. Some formulae have almost no antioxidant effects but may act as pro-oxidants.

## Introduction

Traditional herbal medicines have attracted much attention as complementary treatments. Kampo, a traditional Japanese herbal medicine, is an established system of alternative or complementary medicine that has demonstrated beneficial treatment effects and reduced side effects.^(1,2)^ Multiple reports have revealed antioxidative effects for Kampo formulae or other herbal extract prescriptions, and the antioxidative potencies of Kampo formulae are now pharmacologically recognized as an important therapeutic mechanism.^(3–8)^ However, most studies have evaluated antioxidative mechanisms using a few substances or stable end products of oxidative reactions. In contrast to the substantial developments in the analysis of post-cellular reactions, the “upstream” aspect of oxidative stress [i.e., the identification of reactive oxygen species (ROS) that stimulate oxidative stress reactions, or interactions among ROS that generate oxidative stimulators] remains largely unclear.^(9,10)^ Because ROS are not uniform, and individual ROS show specific characteristics during *in vivo* reactions,^(11)^ analysis of multiple ROS is clearly necessary. To address this problem, we have developed an electron spin resonance (ESR)-based method to analyze multiple ROS scavenging activities (multiple free radical scavenging activities: MULTIS) in biological samples and have used it to analyze the upstream aspect of oxidative stress-related reactions.^(12,13)^

This study aimed to investigate the multidimensional antioxidative profiles of Kampo formulae by analyzing their relations to clinical effects, and to provide explanations of their Kampo or oriental medical therapeutic effects using Western medical terms. We determined the multiple ROS scavenging activities of 48 clinically used Kampo prescription formulae for prescription. The measured ROS were hydroxyl radical (^•^OH), alkoxyl radical (RO^•^, *tert*-BuO^•^), alkylperoxyl radical (ROO^•^, *tert*-BuOO^•^), superoxide (O_2_^•−^), and singlet oxygen (^1^O_2_). We also analyzed the effects of crude drugs on the antioxidative activities of Kampo formulae. Our findings demonstrate previously unknown antioxidative effects in multiple Kampo formulae.

## Materials and Methods

### Kampo extract preparation

We used 48 Kampo extract prescription formulae widely used in Japan; these are shown in Table 1 and 2. The Kampo extract preparations were donated by Tsumura Co., Ltd (Tokyo, Japan). All preparations were clinical grade extract granules. In clinical use, these extract granules are easily dissolved in approximately 200 ml of hot water or orally administrated as they are. In this study, 2.5 g of the Kampo extract granules were dissolved in ultrapurified water at 90°C and subjected to ESR measurement in 10% aqueous solutions. The relations between ROS scavenging activity and quantity of crude drug components for Kampo preparations were statistically analyzed.

### Measurements of multiple free radical scavenging activity

MULTIS were measured using a previously described ESR-based method with minor modifications.^(12,13)^ MULTIS measurements were performed three times per each ROS. The ESR spectrometer used was a RR-X1 equipped with 100 kHz field modulation and WIN-RAD operation software (Radical Research Inc., Tokyo, Japan). The compound 5-(2,2-dimethyl-1,3-propoxy cyclophosphoryl)-5-methyl-1-pyrroline *N*-oxide (CYPMPO) was used as an ESR spin-trapping reagent. Typical spectrometer settings were field modulation width 0.1 mT; microwave power 10 mW; field scan width and rate ±7.5 mT/2 min; and time constant 0.1 s. Each ROS was produced via *in situ* illumination with UV/visible light from an illuminator (RUVF-203SR UV illuminator; Radical Research Inc., Tokyo, Japan) equipped with a 200 W medium-pressure mercury/xenon arc lamp and a quartz light-guide connected to the resonator cavity. Table 3 summarizes the light sources, illumination times, precursors, and photosensitizers used to produce ROS. ROS scavenging activities were calculated according to a previously described method and expressed as the unit equivalent to known pure scavengers: glutathione (GSH) for ^•^OH and ^1^O_2_, superoxide dismutase (SOD) for O_2_^•−^, 6-hydroxy-2,5,7,8-tetramethylchroman-2-carboxylic acid (TROLOX) for RO^•^, and α-lipoic acid for ROO^•^.^(13)^ To evaluate overall scavenging activity, scavenging activities of each ROS were expressed as relative values with the Kampo formulation tsudosan (TDS) as 1. The scavenging activities were expressed in terms of their daily doses. All samples were prepared in triplicate. Data were expressed as mean values.

### Reagents

CYPMPO was obtained from Radical Research Inc. (Tokyo, Japan); hydrogen peroxide, riboflavin, 2,2'-azobis (2-amidinopropane) hydrochloride (AAPH), *tert*-butyl hydroperoxide, dimethyl sulfoxide (DMSO), rose bengal, and 4-hydroxy-2,2,6,6-tetramethylpiperidine (4-OH-TMPO) were purchased from Tokyo Chemical Industry (Tokyo, Japan) and used without modification. Buffers and biochemical reagents were obtained from Wako Chemical Co. (Osaka, Japan).

### Statistical analysis

Statistical analysis was performed using computer software (Prism 6; GraphPad Software Inc., La Jolla, CA). Data were tested using Spearman’s rank correlation coefficient.

## Results

### Hydroxyl radical scavenging activity of Kampo formulae

**Fig. 1:**
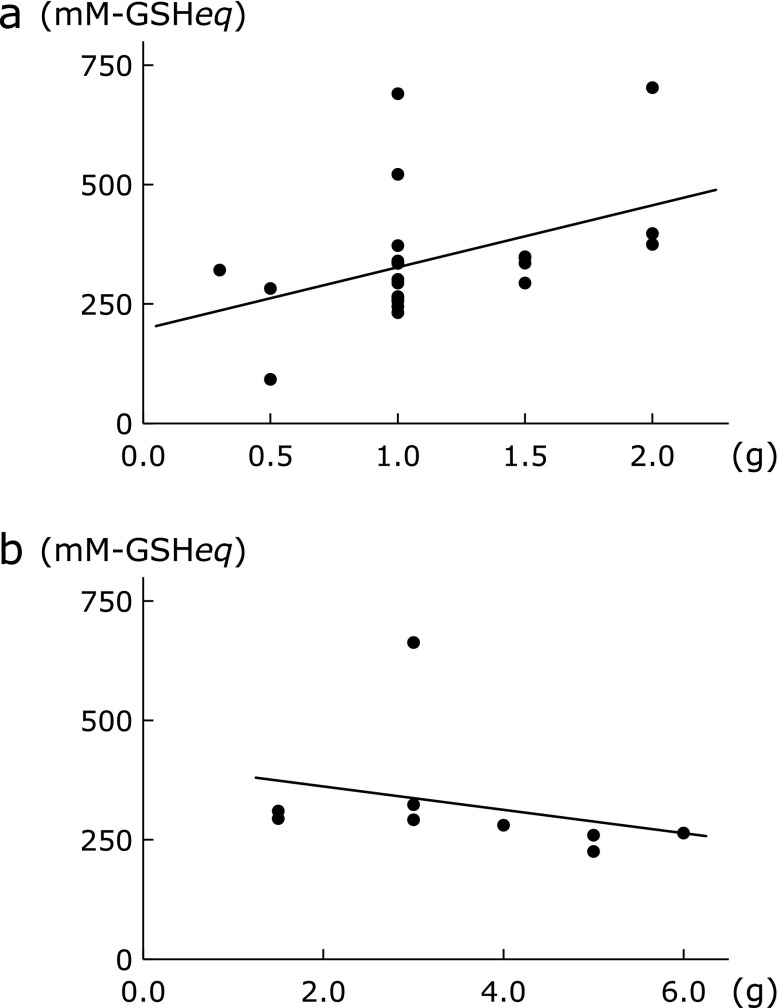
Correlation between hydroxyl radical scavenging activity and the amount of *Zingiberis Rhizoma* (a) and *Rehmanniae Radix* (b). *r* = 0.54, *p*<0.05 for *Zingiberis Rhizoma* and *r* = −0.75, *p*<0.05 for *Rehmanniae Radix*.

Table 4 shows the ^•^OH scavenging activity of Kampo extract preparations. The formulae shosaikoto (SSK), saikokaryukotsuboreito (SRB), unseiin (USI), and daisaikoto (DST) showed remarkably high ^•^OH scavenging activity. Among them, shosaikoto (SSK), saikokaryukotsuboreito (SRB), and daisaikoto (DST) contain both *Bupleuri Radix* and *Scutellariae Radix* as components, which may relate to antioxidative activity [the amount of *Bupleuri Radix* and *Scutellariae Radix* were, respectively, 7 g and 3 g in shosaikoto (SSK), 5 g and 2.5 g in saikokaryukotsuboreito (SRB), and 6 g and 3 g in daisaikoto (DST), per daily dose]. *Zingiberis Rhizoma* showed a significant positive correlation and *Rehmanniae Radix* showed a significant strong negative correlation between ^•^OH scavenging activity and quantity (*r* = 0.54, *p*<0.05 for *Zingiberis Rhizoma* and *r* = −0.75, *p*<0.05 for *Rehmanniae Radix*, Fig. 1).

### Alkoxyl radical scavenging activity of Kampo formulae

**Fig. 2:**
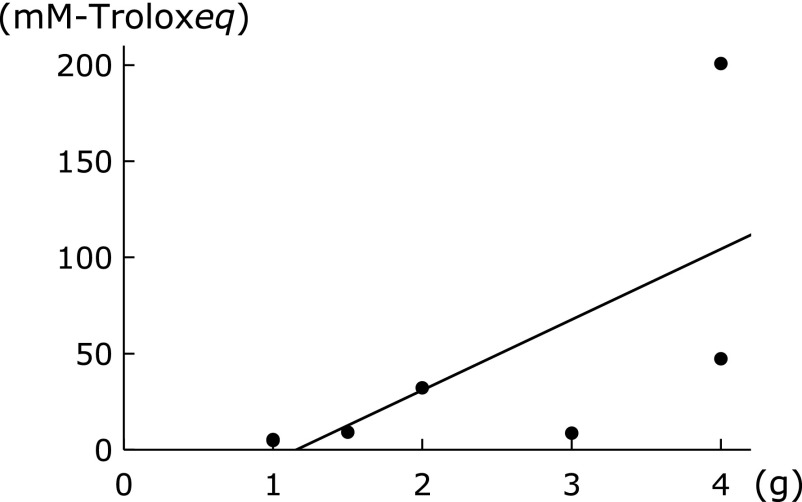
Correlation between alkoxyl radical scavenging activity and the amount of *Rhei Rhizoma* (*r* = 0.87, *p*<0.05).

Table 5 shows the RO^•^ scavenging activity of Kampo formulae extracts. Daiokanzoto (DKZT), which contains large amounts of *Rhei Rhizoma* (4 g per daily dose in the extract), showed remarkably high RO^•^ scavenging activity. There was a significant strong positive correlation between the amount of *Rhei Rhizoma* and RO^•^ scavenging activity (*r* = 0.87, *p*<0.05, Fig. 2). Other formulae containing *Rhei Rhizoma*, including mashiningan (MNG) and daiobotanpito (DBT), also showed high RO^•^ scavenging activity [the amount of *Rhei Rhizoma* was 4 g in mashiningan (MNG) and 2 g in daiobotanpito (DBT) per daily dose in the extract]. Formulae that included *Coptidis Rhizoma*, namely orengedokuto (OGT), unseiin (USI), and saikoseikanto (SSET), also showed high RO^•^ scavenging activity; however, the results were not significant owing to the small number of formulae that contained the component. A previous report found that Kampo formulae containing *Rhei Rhizoma* showed high oxygen radical absorbance capacity (ORAC) values;^(3)^ our results for RO^•^ scavenging activity generally agree with these previous findings.

### Alkylperoxyl radical scavenging activity of Kampo formulae

**Fig. 3:**
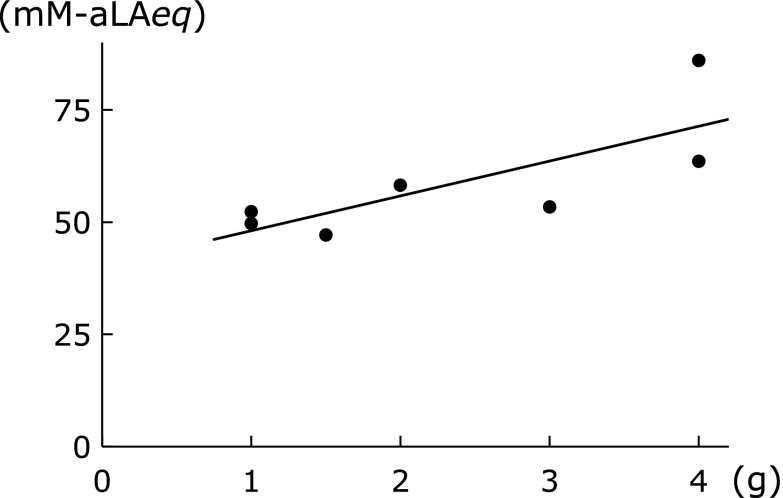
Correlation between alkylperoxyl radical scavenging activity and the amount of *Rhei Rhizoma* (*r* = 0.84, *p*<0.05).

Table 6 shows the ROO^•^ scavenging activity of Kampo formulae extracts. The highest ROO^•^ scavenging activity was found in kakkontokasenkyushin'i (KTSS) and maoto (MT), both of which contain *Ephedrae Herba*. However, this component had no significant effect on ROO^•^ scavenging activity. The only crude element that significantly contributed to ROO^•^ scavenging activity was *Rhei Rhizoma* (*r* = 0.84, *p*<0.05, Fig. 3). Instead, ROO^•^ scavenging activity was notable in saibokuto (SBT), hangekobokuto (HKT), and goreisan (GRS), whose antioxidative activities were previously unknown. Moreover, many of our results related to ROO^•^ scavenging activity could not be explained by the constitution of herbal ingredients.

### Superoxide scavenging activity of Kampo formulae

Table 7 shows the O_2_^•−^ scavenging activity of Kampo formulae extracts. Formulae containing *Scutellariae Radix*, *Phellodendri Cortex*, and* Coptidis Rhizoma*, namely orengedokuto (OGT), unseiin (USI), and keigairengyoto (KRT), showed high scavenging activity against O_2_^•−^ [the amounts of *Scutellariae Radix*, *Phellodendri Cortex* and *Coptidis Rhizoma* were, respectively, 3 g, 1.5 g and 2 g in orengedokuto (OGT); 1.5 g, 1.5 g and 1.5 g in unseiin (USI); and 1.5 g, 1.5 g and 1.5 g in keigairengyoto (KRT) per daily dose]. High O_2_^•−^ scavenging activity was also found in formulae containing *Rhei Rhizoma* [i.e., daiokanzoto (DKZT) and tsudosan (TDS)] and *Ephedrae Herba* [i.e., kakkontokasenkyushin'i (KTSS) and maoto (MT)]. Again, no significant results were observed owing to the small number of formulae that contained the component.

### Singlet oxygen scavenging activity of Kampo formulae

**Fig. 4:**
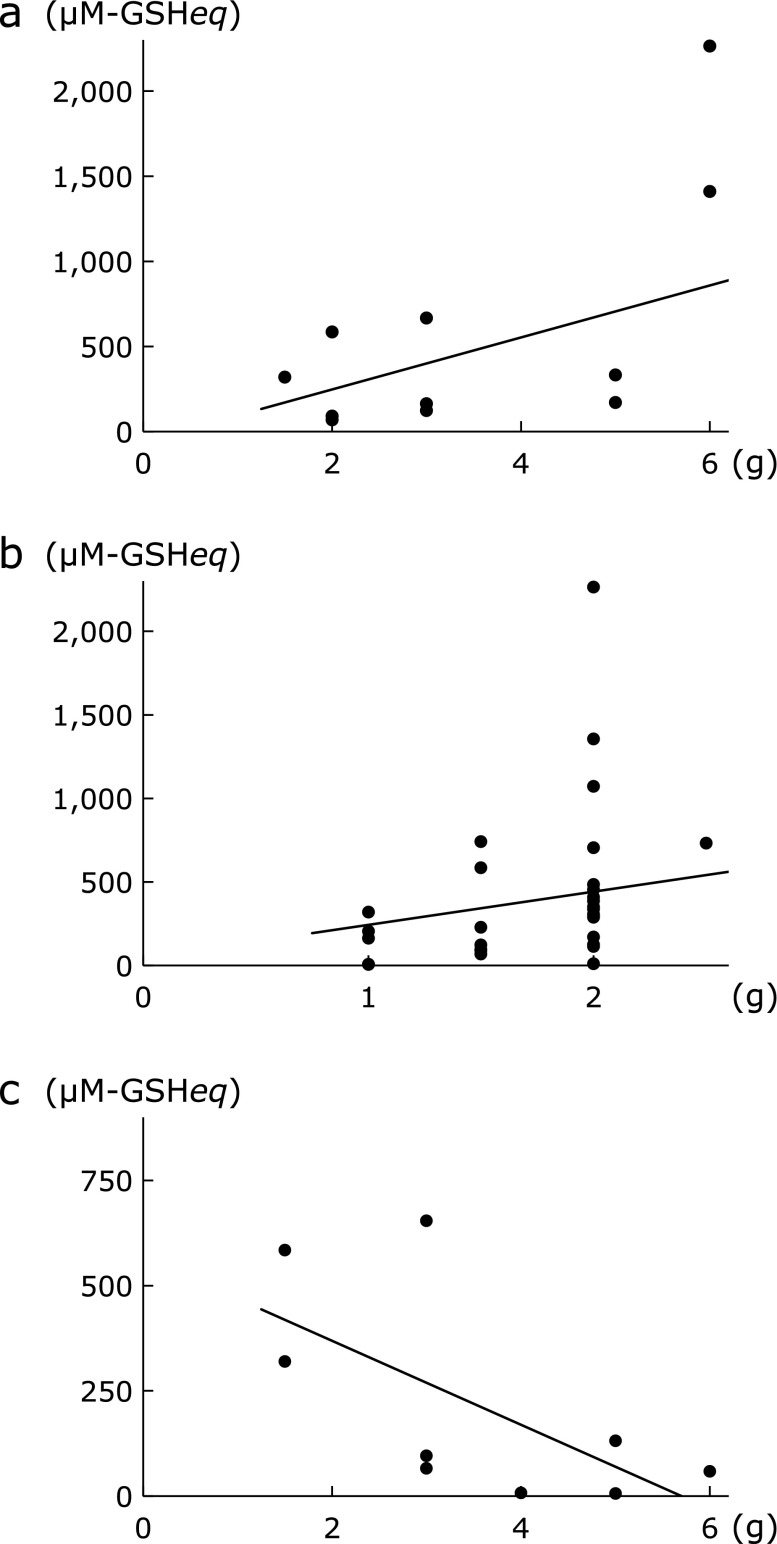
Correlation between singlet oxygen scavenging activity and the amount of *Bupleuri Radix* (a) *r* = 0.57, *p*<0.05; *Glycyrrhizae Radix* (b) *r* = 0.40, *p*<0.05; and *Rehmanniae Radix* (c) *r* = −0.67, *p*<0.05).

Table 8 lists the Kampo formulae that showed high ^1^O_2_ scavenging activity of extracts. Formulae containing *Bupleuri Radix*, namely saikokeishikankyoto (SAKK), daisaikoto (DST), shosaikoto (SSK), saibokuto (SBT) and saikoseikanto (SSET), showed high scavenging activity against ^1^O_2_ [the amount of *Bupleuri Radix* was 6 g in saikokeishikankyoto (SAKK), 6 g in daisaikoto (DST), 7 g in shosaikoto (SSK), 7 g in saibokuto (SBT) and 6 g in saikoseikanto (SSET) per daily dose] and there was a significant positive correlation between the quantity of *Bupleuri Radix* and ^1^O_2_ scavenging activity (*r* = 0.57, *p*<0.05, Fig. 4a). The quantity of *Glycyrrhizae Radix* was also positively correlated with ^1^O_2_ scavenging activity (*r* = 0.40, *p*<0.05, Fig. 4b).

In contrast, some formulae showed remarkably low ^1^O_2_ scavenging activity. The amount of *Rehmanniae Radix* showed a negative correlation with ^1^O_2_ scavenging activity (*r* = −0.67, *p*<0.05, Fig. 4c), a finding similar to the results for ^•^OH scavenging activity. Formulae containing high doses of *Rehmanniae Radix*, namely goshajinkigan (GJG), tokiinshi (TKI) and hachimijiogan (HJG), showed generally low ^1^O_2_ scavenging activity. Furthermore, *Atractylodis Rhizoma*, *Processi Aconiti Radix*, and *Zingiberis Processum Rhizoma* also showed a tendency to decrease ^1^O_2_ scavenging activity, although these results were not statistically significant (data not shown).

### Overall scavenging activities against multiple ROS

**Fig. 5:**
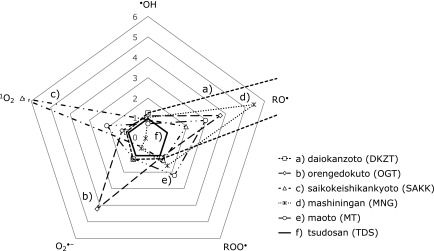
Total ROS scavenging profile of representative Kampo formulae, namely daiokanzoto (DKZT) (a), orengedokuto (OGT) (b), saikokeishikankyoto (SAKK) (c), mashiningan (MNG) (d), maoto (MT) (e), and tsudosan (TDS) (f). The scavenging activities against each ROS are expressed as relative values in comparison with tsudosan.

Figure 5 summarizes the total ROS scavenging profile of five representative Kampo formulae that exhibited high scavenging activity against each ROS compared with tsudosan (TDS), the formula reported to possess the highest ORAC value.^(3)^ Formulae containing high amounts of *Rhei Rhizoma*, daiokanzoto (DKZT) and mashiningan (MNG) showed solid RO^•^ and ROO^•^ scavenging activities. The RO^•^ scavenging activity of these two formulae was remarkably strong even when they were compared with tsudosan (TDS). The ROS scavenging activities of orengedokuto (OGT), which contains *Scutellariae Radix*, *Phellodendri Cortex* and *Coptidis Rhizoma*, were remarkable against O_2_^•−^. Saikokeishikankyoto (SAKK), a representative formula of a prescription containing *Bupleuri Radix*, showed high ^1^O_2_ scavenging activity.

## Discussion

Because ROS possess high reactivity and their reactions are varied and complicated, it is difficult to describe the details of these pathways. This uncertainty is an obstacle for clinical antioxidative therapy and an analysis of multiple ROS pathways is strongly needed.^(10)^ Typically, an oxidative stress-related reaction is initiated by an excess production of primary ROS leading to further radical chain reactions, which then evoke cellular antioxidative responses. It is mainly superoxide that has this initiative role in biological systems. Thereafter, radical chain reactions involving ^•^OH are evoked, leading to lipid hydroperoxide (LOOH) production. In the presence of iron, copper, or heme protein, LOOH is converted to lipid alkoxyl radical (LO^•^) or lipid alkylperoxyl radical (LOO^•^), leading to further radical chain reactions. ^1^O_2_ also plays an important role in LOOH production.^(14)^ Thus, each ROS shows specific characteristics during *in vivo* reactions.^(11)^ Because Kampo or other traditional herbal prescriptions contain various active ingredients, the evaluation of multiple ROS dynamics is necessary for detailed analysis of their redox activity.

Oxidative stress is a critical and universal pathological factor that impacts various diseases. Antioxidative effects are recognized as the most important therapeutic mechanism of Kampo formulae. Many studies have demonstrated antioxidative activities for Kampo formulae and other herbal medicines, including their crude components,^(3–7)^ but many details about the mechanisms of these activities remain to be clarified. Our results revealed that Kampo formulae known to present high ORAC values,^(3)^ such as mashiningan (MNG) and daiokanzoto (DKZT), possess high RO^•^ scavenging activity. We also found that *Rhei Rhizoma*, a key crude drug that shows high ORAC values, showed a positive correlation between its quantity in the formulae and RO^•^ scavenging activity. Therefore, much of the previously demonstrated antioxidative activity of Kampo formulae reflects RO^•^ scavenging activity.

^•^OH is considered the most toxic oxygen radical because of its extremely high reactivity against biological substances, leading to tissue/organ damage. No specific substances eliminate ^•^OH *in vivo*; however, ^•^OH scavenging activity is strongly related to the pathophysiology of multiple diseases.^(13,15,16)^ Kampo formulae containing *Zingiberis Rhizoma* showed high scavenging activity against ^•^OH. *Zingiberis Rhizoma* contains multiple pharmacologically active components, including zingiberol and shogaol, and exhibits various pharmacological effects.^(17)^ Although *Zingiberis Rhizoma* shows low antioxidative activity against AAPH-induced oxidative stress,^(18)^ our findings revealed remarkable ^•^OH scavenging activity in formulae containing *Zingiberis Rhizoma*. Some of its pharmacological effects may be induced by high ^•^OH scavenging activity. In addition, formulae containing both *Bupleuri Radix* and *Scutellariae Radix*, namely shosaikoto (SSK), saikokaryukotsuboreito (SRB), and daisaikoto (DST), showed strong ^•^OH scavenging activity [but saibokuto (SBT) did not]. Clinically, *Bupleuri Radix* and *Scutellariae Radix* are frequently used in combination and this combination shows stronger anti-inflammatory effects than single usage.^(19)^ Among the formulae used in this study, 69% of formulae containing *Scutellariae Radix* also contain *Bupleuri Radix* and their high ^•^OH scavenging activity provides a pharmacological explanation for the traditional use of the crude composition.

We measured the scavenging activity of ROO^•^ using *tert*-BuOOH; thus, this activity might reflect the lipid peroxidation process. Again, details of the activities of ROO^•^ remain to be clarified, but current research indicates its importance in an animal model of hepatic carcinoma.^(20)^ Unlike the other ROS, ROO^•^ scavenging activity was notable in formulae whose antioxidative activities were previously unknown, such as saibokuto (SBT), hangekobokuto (HKT) and goreisan (GRS). Because saibokuto (SBT) is a blended formula comprising hangekobokuto (HKT) and shosaikoto (SSK), the components of hangekobokuto (HKT) may relate to ROO^•^ scavenging activity. As there were a small number of formulations and many combinations of crude drug ingredients, it was difficult to identify specific important responsible substances. Many results related to ROO^•^ scavenging activity could not be explained by the constitution of herbal ingredients.

The superoxide scavenging activity of Kampo formulae has been widely reported.^(21–26)^ A series of studies by Kohno and colleagues report an extensive screening of O_2_^•−^ scavenging activity of herbal extracts.^(4,5)^ They found that extracts such as *Rheum palmatum*, *Ephedra sinica*, *Punica granatum* and *Caesalpinia sappan* show high O_2_^•−^ scavenging activity. In our analysis of the formulations, we could not obtain a single crude component that enhanced O_2_^•−^ scavenging activity. However, formulae containing the three crude extracts *Scutellariae Radix*, *Coptidis Rhizoma* and *Phellodendri Cortex* [i.e., orengedokuto (OGT), unseiin (USI) and keigairengyoto (KRT)] showed remarkably high O_2_^•−^ scavenging activity. Consequently, in contrast to the ^•^OH scavenging activity of *Scutellariae Radix*, the combination of these three crude extracts may be crucial for O_2_^•−^ control. Moreover, though unseiin (USI) contains *Rehmanniae Radix*, which decreases multiple ROS scavenging activities (as described below), O_2_^•−^ scavenging activity was still high. Therefore, the combination of *Scutellariae Radix*, *Coptidis Rhizoma* and *Phellodendri Cortex* may recover the pro-oxidative effect of *Rehmanniae Radix*.

^1^O_2_ shows high reactivity and toxicity *in vivo* and leads to lipid peroxidation, inducing further RO^•^ and ROO^•^ production.^(14)^ ^1^O_2_ is produced through myeloperoxidase and prostaglandin hydroperoxidase activity, and by the interaction between O_2_^•−^ and H_2_O_2_ during the Haber-Weiss reaction.^(27,28)^ The importance of ^1^O_2_ has been shown in several important pathophysiological processes, including ischemia-reperfusion injury and diabetes.^(29)^ We previously reported a crucial change of *in vivo* ^1^O_2_ scavenging activity after an administration of kangen-karyu, a Kampo formula; however, the importance of this activity in Kampo or other herbal medicine remains to be clarified. High ^1^O_2_ scavenging activity was detected in the formulae containing *Bupleuri Radix*; that is, saikokeishikankyoto (SAKK), daisaikoto (DST) and shosaikoto (SSK). The quantity of *Bupleuri Radix* showed a significant positive correlation with ^1^O_2_ scavenging activity. Thus, *Bupleuri Radix* is responsible for both ^•^OH and ^1^O_2_ scavenging activity. In contrast, it is notable that some formulae showed remarkably low ^1^O_2_ scavenging activity. In particular, the ^1^O_2_ scavenging activity of tokiinshi (TKI), kamikihito (KT) and goshajinkigan (GJG) was less than 1/200 of that of saikokeishikankyoto (SAKK).

Interestingly, the quantity of *Rehmanniae Radix* showed a significant negative correlation with ^•^OH and ^1^O_2_ scavenging activities. Furthermore, formulae containing *Rehmanniae Radix* [i.e., hachimijiogan (HJG), goshajinkigan (GJG), rokumigan (RUG) and tokiinshi (TKI)] generally revealed low ROS scavenging activities against ^•^OH, RO^•^, ROO^•^ and ^1^O, but unseiin (USI) did not. Thus, *Rehmanniae Radix* may act as a pro-oxidant, rather than an antioxidant. Clinically, formulae that include *Rehmanniae Radix* are used in “*hohou*.” *Hohou* is a revitalizing treatment that stimulates or compensates functions that are lacking and is used to treat chronic diseases, exhaustion, or aging, conditions in which pathophysiology is strongly related to oxidative stress. Kampo emphasizes the concept of equilibrium, which is similar to homeostasis in Western medicine; the concept of oxidative stress is based on the assumption of a pro-oxidant–antioxidant equilibrium.^(30)^ Thus, treatment of oxidative stress by Kampo medicine aims to rebalance the oxidative–antioxidative equilibrium (which is assumed to be tilted toward oxidation) by supplementing antioxidative crude extracts or elements. However, our findings of the low ROS scavenging activities of *Rehmanniae Radix*, which is a representative crude extract in *hohou* formulae, totally contradicts this assumption. Therefore, we hypothesize that Kampo oxidative stress treatments do not simply complement antioxidants, but induce internal antioxidative activity by stimulating pro-oxidative elements.

As the “antioxidant paradox” concept indicates, it remains unclear whether treatment of oxidative stress by administration of antioxidants benefits the whole body.^(10)^ Rather, it is highly likely that weak pro-oxidants have better effects on living bodies than antioxidants, through a radiation hormesis-like beneficial effect.^(31)^ However, it is currently difficult to obtain approval for clinical trials using pro-oxidants owing to ethical problems. Kampo is the most systematically organized complementary and alternative medical treatment. Its safety has been established and it is widely used. Therefore, elucidation of the pro-oxidative effects of Kampo might be a promising yet realistic research pathway toward the therapeutic application of pro-oxidants. Further investigations that include oxidative stress evaluations may lead to a new concept of oxidative stress treatment using pro-oxidants.

## Figures and Tables

**Table 1:** List of Kampo formulae and their abbreviations

Name	Abbr.	Name	Abbr.	Name	Abbr.
bakumondoto	BAK	kakkontokasenkyushin'i	KTSS	saikokeishito	SAKT
bofutsushosan	BTS	kamikihito	KKT	saikoseikanto	SSET
boiohito	BOT	kamishoyosan	KSS	saireito	SRT
daikenchuto	DKT	keigairengyoto	KRT	shimbuto	SIT
daiobotanpito	DBT	keishibukuryogan	KBG	shimotsuto	SMT
daiokanzoto	DKZT	keishikajutsubuto	KSTJB	shokenchuto	SHKT
daisaikoto	DST	keishikaryukotsuboreito	KSTRB	shosaikoto	SSK
goreisan	GRS	keishito	KST	shoseiryuto	SST
goshajinkigan	GJG	maoto	MT	tokiinshi	TKI
goshuyuto	GSY	mashiningan	MNG	tokishakuyakusan	TSS
hachimijiogan	HJG	ninjinto	NT	tokishigyakukagoshuyushokyoto	TSGST
hangekobokuto	HKT	orengedokuto	OGT	tsudosan	TDS
hangeshoshinto	HST	rikkunshito	RKT	unseiin	USI
hochuekkito	HET	rokumigan	RJG	yokuininto	YT
jumihaidokuto	JHT	saibokuto	SBT	yokukansan	YKS
juzentaihoto	JTT	saikokaryukotsuboreito	SRB		
kakkonto	KT	saikokeishikankyoto	SAKK		

Abbr.: abbreviation

**Table 2:** List of Kampo formulae and their composition of crude extracts. The crude amounts are indicated as daily dose

Name		Major components (gram per daily dose)												
	Abbreviation	Scutellariae Radix	Phellodendri Cortex	Coptidis Rhizoma	Zingiberis Processum Rhizoma	Glycyrrhizae Radix	Bupleuri Radix	Rehmanniae Radix	Paeoniae Radix	Zingiberis Rhizoma	Rhei Rhizoma	Atractylodes Lancea Rhizome	Processi Aconiti Radix	Ephedrae Herba
bakumondoto	BAK					2								
bofutsushosan	BTS	2				2			1.2	0.3	1.5			1.2
boiohito	BOT					1.5				1		3		
daikenchuto	DKT				5									
daiobotanpito	DBT										2			
daiokanzoto	DKZT					2					4			
daisaikoto	DST	3					6		3	1	1			
goreisan	GRS											3		
goshajinkigan	GJG							5					1	
goshuyuto	GSY									1.5				
hachimijiogan	HJG							6					0.5	
hangekobokuto	HKT									1				
hangeshoshinto	HST	2.5		1	2.5	2.5								
hochuekkito	HET					1.5	2			0.5		4		
jumihaidokuto	JHT					1	3			1				
juzentaihoto	JTT					1.5		3	3			3		
kakkonto	KT					2			2	2				3
kakkontokasenkyushin'i	KTSS					2			2	2				3
kamikihito	KKT					1	3			1		3		
kamishoyosan	KSS					1.5	3		3	1		3		
keigairengyoto	KRT	1.5	1.5	1.5		1	1.5	1.5	1.5					
keishibukuryogan	KBG								3					
keishikajutsubuto	KSTJB					2			4	1		4	0.5	
keishikaryukotsuboreito	KSTRB					2			4	1.5				
keishito	KST					2			4	1.5				
maoto	MT					1.5								5
mashiningan	MNG								2		4			
ninjinto	NT				3	3						3		
orengedokuto	OGT	3	1.5	2										
rikkunshito	RKT					1				0.5		4		
rokumigan	RJG							5						
saibokuto	SBT	3				2	7			1				
saikokaryukotsuboreito	SRB	2.5					5			1	1			
saikokeishikankyoto	SAKK	3			2	2	6							
saikokeishito	SAKT	2				2	5		2	1				
saikoseikanto	SSET	1.5	1.5	1.5		1.5	2	1.5	1.5					
saireito	SRT	3				2	7			1		3		
shimbuto	SIT								3	1.5		3	0.5	
shimotsuto	SMT							3	3					
shokenchuto	SHKT					2			6	1				
shosaikoto	SSK	3				2	7			2				
shoseiryuto	SST				3	3			3					3
tokiinshi	TKI					1		4	3					
tokishakuyakusan	TSS								4			4		
tokishigyakukagoshuyushokyoto	TSGST					2			3	1				
tsudosan	TDS					2					3			
unseiin	USI	1.5	1.5	1.5				3	3					
yokuininto	YT					2			3			4		4
yokukansan	YKS					1.5	2					4		

**Table 3:** Photolytic production methods of multiple reactive oxygen species

Free radical	Spin trap	Precursor/Sensitizer	UV/VL	Irradiation period	Antioxiidant equivalent
^•^OH	CYPMPO	H_2_O_2_ 10 mM	UV	5 s	GSH
O_2_^•−^	CYPMPO	Riboflavin 20 µM	VL	60 s	SOD
RO^•^	CYPMPO	AAPH 10 mM	UV	5 s	Trolox
ROO^•^	CYPMPO	*tert*-BHP 10 mM	UV	5 s	α-lipoic acid
^1^O_2_	TMPO	Rosebengal 200 µM	VL	30 s	GSH

UV, ultraviolet (300–400 nm); VL, visual light (500–600 nm); CYPMPO, 5-(2,2-dimethyl-1,3-propoxy cyclophosphoryl)-5-methyl-1-pyrroline *N*-oxide; AAPH, 2,2'-azobis (2-amidinopropane) hydrochloride; *tert*-BHP, *tert*-butyl hydroperoxide; GSH, glutathione; SOD, superoxide dismutase; TMPO, 4-hydroxy-2,2,6,6-tetramethylpiperidine.

**Table 4:** Hydroxyl radical scavenging activity of Kampo formulae

Kampo formula	^•^OH scavenging activity (mM-GSH*eq*)	
	per daily dose	per gram
shosaikoto	703.1	93.7
saikokaryukotsuboreito	690.3	92.0
unseiin	663.0	88.4
daisaikoto	521.8	69.6
kakkonto	397.3	53.0
daiokanzoto	393.7	52.5
bakumondoto	382.2	42.5
ninjinto	376.0	50.1
kakkontokasenkyushin'i	375.0	50.0
saikokeishito	372.3	49.6
orengedokuto	349.6	46.6
keishito	348.9	46.5
keishikaryukotsuboreito	347.0	46.3
keishikajutsubuto	339.8	45.3
goreisan	339.8	45.3
boiohito	336.8	44.9
shimbuto	335.6	44.7
hangekobokuto	335.1	44.7
mashiningan	325.1	43.3
jyuzendaihoto	323.9	43.2
bofutsushosan	321.3	42.8
saikoseikanto	310.4	41.4
tsudosan	308.5	41.1
daiobotanbito	301.2	40.2
jumihaidokuto	301.2	40.2
yokkansan	296.8	39.6
keigairengyoto	294.3	39.2
shokenchuto	294.2	19.6
goshuyuto	294.0	39.2
shimotsuto	292.3	39.0
saikokeishikankyoto	292.2	39.0
hangeshashinto	292.1	38.9
rikkunshito	282.5	37.7
tokiinshi	280.4	37.4
kamishoyosan	265.7	35.4
hachimijiogan	264.1	35.2
kamikihito	261.2	34.8
goshajinkigan	259.6	34.6
tokishigyakukagoshuyushokyoto	255.7	34.1
tokishakuyakusan	251.2	33.5
keishibukuryogan	250.2	33.4
saibokuto	244.4	32.6
daikenchuto	242.1	16.1
saireito	232.3	25.8
yokuininto	231.8	30.9
maoto	231.6	30.9
rokumigan	225.7	30.1
shoseiryuto	98.4	13.1
hochuekkito	92.2	12.3

**Table 5:** Alkoxyl radical scavenging activity of Kampo formulae

Kampo formula	RO^•^ scavenging activity (mM-TROLOX*eq*)	
	per daily dose	per gram
daiokanzoto	200.7	26.8
mashiningan	47.3	6.3
orengedokuto	32.3	4.3
daiobotanbito	32.2	4.3
maoto	25.7	3.4
saikokeishikankyoto	17.0	2.3
unseiin	14.9	2.0
yokuininto	12.5	1.7
saikoseikanto	11.9	1.6
bofutsushosan	9.2	1.2
kakkontokasenkyushin'i	9.2	1.2
tsudosan	8.7	1.2
keigairengyoto	8.4	1.1
hangeshashinto	6.8	0.9
keishibukuryogan	6.5	0.9
saibokuto	5.9	0.8
daisaikoto	5.3	0.7
hochuekkito	4.8	0.6
hangekobokuto	4.8	0.6
saikokaryukotsuboreito	4.8	0.6
ninjinto	4.5	0.6
kakkonto	4.2	0.6
daikenchuto	4.2	0.3
shosaikoto	4.2	0.6
shokenchuto	4.1	0.3
yokkansan	4.1	0.5
saikokeishito	3.9	0.5
keishito	3.8	0.5
saireito	3.7	0.4
keishikaryukotsuboreito	3.5	0.5
keishikajutsubuto	3.1	0.4
boiohito	3.1	0.4
goshuyuto	3.1	0.4
rokumigan	3.0	0.4
tokiinshi	2.8	0.4
jyuzendaihoto	2.7	0.4
rikkunshito	2.6	0.3
kamishoyosan	2.5	0.3
kamikihito	2.4	0.3
shimbuto	2.4	0.3
goshajinkigan	2.3	0.3
shimotsuto	2.3	0.3
shoseiryuto	2.2	0.3
jumihaidokuto	1.8	0.2
bakumondoto	1.8	0.2
tokishigyakukagoshuyushokyoto	1.7	0.2
tokishakuyakusan	1.7	0.2
goreisan	1.4	0.2
hachimijiogan	0.9	0.1

**Table 6:** Alkylperoxyl radical scavenging activity of Kampo formulae

Kampo formula	ROO^•^ scavenging activity (mM-aLA*eq*)	
	per daily dose	per gram
kakkontokasenkyushin'i	148.4	19.8
maoto	117.4	15.7
keishikaryukotsuboreito	90.8	12.1
saibokuto	89.6	12.0
hangekobokuto	86.3	11.5
mashiningan	86.0	11.5
hochuekkito	85.6	11.4
goreisan	81.1	10.8
keishikajutsubuto	79.0	10.5
saikokeishito	76.9	10.2
yokkansan	74.1	9.9
jyuzendaihoto	72.5	9.7
unseiin	71.8	9.6
ninjinto	71.7	9.6
yokuininto	70.6	9.4
keigairengyoto	69.8	9.3
saikokeishikankyoto	68.8	9.2
daiokanzoto	63.5	8.5
keishito	62.8	8.4
orengedokuto	59.1	7.9
daiobotanbito	58.2	7.8
saikoseikanto	54.7	7.3
rokumigan	53.5	7.1
tsudosan	53.4	7.1
kamikihito	53.4	7.1
shosaikoto	52.8	7.0
saireito	52.8	5.9
saikokaryukotsuboreito	52.4	7.0
jumihaidokuto	51.3	6.8
goshuyuto	50.3	6.7
daisaikoto	49.7	6.6
shimbuto	49.7	6.6
kamishoyosan	49.3	6.6
keishibukuryogan	49.0	6.5
goshajinkigan	48.7	6.5
hachimijiogan	48.0	6.4
tokishakuyakusan	47.9	6.4
tokiinshi	47.5	6.3
bofutsushosan	47.1	6.3
boiohito	46.6	6.2
rikkunshito	44.1	5.9
tokishigyakukagoshuyushokyoto	42.9	5.7
daikenchuto	42.6	2.8
shimotsuto	42.4	5.7
shokenchuto	41.8	2.8
hangeshashinto	41.0	5.5
kakkonto	41.0	5.5
bakumondoto	36.5	4.1
shoseiryuto	34.6	4.6

**Table 7:** Superoxide scavenging activity of Kampo formulae

Kampo formula	O_2_^•−^ scavenging activity (U/ml-SOD*eq*)	
	per daily dose	per gram
orengedokuto	3,522.3	469.6
saikokaryukotsuboreito	2,499.8	333.3
unseiin	2,321.0	309.5
keigairengyoto	1,482.9	197.7
yokkansan	1,400.1	186.7
kakkontokasenkyushin'i	1,107.3	147.6
saireito	1,036.4	115.2
daiokanzoto	1,023.3	136.4
maoto	991.3	132.2
tokiinshi	834.1	111.2
tsudosan	833.5	111.1
saibokuto	751.0	100.1
goshajinkigan	704.1	93.9
shokenchuto	698.5	46.6
daisaikoto	661.6	88.2
kamikihito	614.7	82.0
rokumigan	604.1	80.5
hangeshashinto	580.5	77.4
hochuekkito	540.2	72.0
kakkonto	538.9	71.9
yokuininto	525.4	70.1
daikenchuto	469.0	31.3
mashiningan	468.5	62.5
keishibukuryogan	454.4	60.6
daiobotanbito	444.4	59.3
shoseiryuto	423.4	56.5
saikokeishikankyoto	406.6	54.2
goshuyuto	394.8	52.6
keishito	382.9	51.0
hangekobokuto	359.8	48.0
shosaikoto	343.1	45.7
ninjinto	333.2	44.4
bofutsushosan	331.8	44.2
shimotsuto	313.5	41.8
boiohito	304.5	40.6
keishikaryukotsuboreito	299.5	39.9
tokishigyakukagoshuyushokyoto	287.8	38.4
hachimijiogan	277.0	36.9
saikoseikanto	269.0	35.9
jumihaidokuto	260.0	34.7
keishikajutsubuto	258.1	34.4
goreisan	251.1	33.5
kamishoyosan	240.0	32.0
saikokeishito	230.9	30.8
rikkunshito	198.4	26.5
jyuzendaihoto	187.4	25.0
shimbuto	179.7	24.0
bakumondoto	161.6	18.0
tokishakuyakusan	143.7	19.2

**Table 8:** Singlet oxygen scavenging activity of Kampo formulae

Kampo formula	^1^O_2_ scavenging activity (µM-GSH*eq*)	
	per daily dose	per gram
saikokeishikankyoto	2,265.4	302.1
daisaikoto	1,410.9	188.1
kakkonto	1,356.2	180.8
shosaikoto	1,071.4	142.9
goshuyuto	977.9	130.4
maoto	742.3	99.0
hangeshashinto	732.4	97.6
saibokuto	705.7	94.1
unseiin	654.6	87.3
saikoseikanto	584.5	77.9
daiobotanbito	515.0	68.7
bofutsushosan	485.7	64.8
keishibukuryogan	475.1	63.4
orengedokuto	467.3	62.3
kakkontokasenkyushin'i	454.0	60.5
hangekobokuto	451.6	60.2
shokenchuto	408.1	27.2
daiokanzoto	386.4	51.5
tsudosan	350.5	46.7
tokishigyakukagoshuyushokyoto	338.5	45.1
saikokaryukotsuboreito	332.5	44.3
keigairengyoto	319.9	42.6
yokuininto	307.9	41.0
saireito	295.9	32.9
keishito	288.1	38.4
ninjinto	269.0	35.9
daikenchuto	243.8	16.3
boiohito	228.9	30.5
rikkunshito	205.7	27.4
shoseiryuto	172.2	23.0
saikokeishito	171.3	22.8
jumihaidokuto	163.1	21.7
rokumigan	131.6	17.5
keishikaryukotsuboreito	125.6	16.8
kamishoyosan	124.4	16.6
keishikajutsubuto	115.0	15.3
jyuzendaihoto	95.9	12.8
hochuekkito	91.1	12.1
shimbuto	82.6	11.0
yokkansan	68.6	9.1
goreisan	66.8	8.9
shimotsuto	66.0	8.8
tokishakuyakusan	65.4	8.7
hachimijiogan	58.7	7.8
mashiningan	40.8	5.4
bakumondoto	10.5	1.2
tokiinshi	8.0	1.1
kamikihito	6.6	0.9
goshajinkigan	6.0	0.8

## Abbreviations

Table 1 shows abbreviations of the Kampo formulae. Because Kampo formulae have complex names, we have included both the formal name of Kampo formulae and their abbreviations throughout the manuscript.

### Abbreviations

AAPH: 2,2'-azobis (2-amidinopropane) hydrochloride
CYPMPO: 5-(2,2-dimethyl-1,3-propoxy cyclophosphoryl)-5-methyl-1-pyrroline *N*-oxide
DMSO: dimethyl sulfoxide
ESR: electron spin resonance
GSH: glutathione
LOOH: lipid hydroperoxide
MULTIS: multiple free radical scavenging activities
4-OH-TMPO: 4-hydroxy-2,2,6,6-tetramethylpiperidine
ORAC: oxygen radical absorbance capacity
ROS: reactive oxygen species
SOD: superoxide dismutase
TROLOX: 6-hydroxy-2,5,7,8-tetramethylchroman-2-carboxylic acid
